# The Human Adenovirus Type 5 L4 Promoter Is Negatively Regulated by TFII-I and L4-33K

**DOI:** 10.1128/JVI.00683-15

**Published:** 2015-04-29

**Authors:** Jordan Wright, Zeenah Atwan, Susan J. Morris, Keith N. Leppard

**Affiliations:** School of Life Sciences, University of Warwick, Coventry, United Kingdom

## Abstract

The late phase of adenovirus gene expression is controlled by proteins made in the intermediate phase, including L4 proteins of 22,000- and 33,000-Da apparent molecular mass (L4-22K and -33K proteins) that are expressed initially from the L4 promoter (L4P). The L4P is activated by a combination of viral proteins and cellular p53 and is ultimately inhibited again by its own products. Here, we have examined the L4P of human adenovirus type 5 in detail and have defined its transcription start site, which our data suggest is positioned by a weak TATA box. Rather than contributing positively to promoter activity, a putative initiator element at the transcription start site acts as a target for negative regulation imposed on the L4P by cellular TFII-I. We show that this TFII-I inhibition is relieved by one of the previously defined viral activators of the L4P, the E4 Orf3 protein, which alters the pool of TFII-I in the cell. We also explore further the negative regulation of the L4P by its products and show that the L4-33K protein is more significant in this process than L4-22K. It is the combined actions of positive and negative factors that lead to the transient activation of the L4P at the onset of the late phase of adenovirus gene expression.

**IMPORTANCE** The adenovirus replication cycle proceeds through multiple phases of gene expression in which a key step is the activation of late-phase gene expression to produce proteins from which progeny particles can be formed. Working with human adenovirus type 5, we showed previously that two proteins expressed from the L4 region of the viral genome perform essential roles in moving the infection on into the late phase; these two proteins are produced by the action of a dedicated promoter, the L4P, and without them the infection does not proceed successfully to progeny generation. In this new work, we delineate further aspects of L4P activity and regulation. Understanding how the L4P works, and how it contributes to activation of the late phase of infection, is important to our understanding of natural infections by the virus, in which late gene expression can fail to occur, allowing the virus to persist.

## INTRODUCTION

During a lytic infection, human adenovirus type 5 (HAdV-C5, referred to here as Ad5) gene expression occurs through a coordinated cascade that begins with expression of early genes and progresses through an intermediate phase to full activation of the virus late genes. The majority of these late genes are part of the major late transcription unit (MLTU), which is divided into five gene blocks, termed L1 to L5, each of which has a single polyadenylation site. The MLTU is controlled by the major late promoter (MLP) and is initially transcribed as a single pre-mRNA that undergoes extensive alternative splicing and polyadenylation to generate the full repertoire of late mRNAs (reviewed in reference 1).

The MLP is weakly active at early times in infection, though expression of genes distal to the L1 unit does not occur (2, 3). Its activity is dramatically upregulated at the onset of the late phase, concomitant with a transition to full expression of L1 to L5 and progressive changes in the splicing pattern within each of the gene blocks (4–6). Two L4 gene products of 22,000- and 33,000-Da apparent molecular mass (L4-22K and L4-33K) have been identified as having crucial roles in the efficient expression of the viral genes within the MLTU (7, 8). L4-22K and L4-33K are N-terminally related proteins that differ in sequence in their carboxyl-terminal domains due to the removal of an intron to form the L4-33K mRNA compared with the L4-22K mRNA (9, 10). L4-22K has been implicated in the activation of the MLP at the onset of the late phase (7, 11, 12), while both L4-33K (13, 14) and L4-22K (7, 11, 15) are required for distinct aspects of the correct and timely splicing of the MLTU pre-mRNA to generate the full repertoire of mature late mRNAs.

The paradoxical requirement for L4-encoded products for the expression of the distal MLTU gene blocks L2 to L5 (i.e., including L4) was resolved by the discovery of a novel promoter termed the L4 promoter (L4P) situated within the L4-100K open reading frame, which drives initial expression of the L4-22K and L4-33K genes (16). Deletion of the L4P from an Ad5 genome leads to decreased and aberrant late gene expression consistent with the loss of L4-22K and L4-33K functions. Thus, the L4P may be considered critical to the early-to-late switch in gene expression that occurs during Ad5 infection, and it is therefore important to determine the regulatory requirements for its induction.

Both viral and cellular proteins have previously been identified as regulators of the L4P. Exogenous expression of the Ad5 E1A, IVa2, and E4 Orf3 proteins was shown to induce L4P activity in luciferase reporter plasmid assays (16). The cellular tumor suppressor p53 has also been identified as a positive regulator of the L4P, and its depletion is inhibitory to both virus late gene expression and the activity of an L4P reporter plasmid (17). Furthermore, endogenous p53 has been found to associate with the L4P around the time of its peak activity during the course of a lytic Ad5 infection (17). However, it is likely that there are other regulators of the L4P that are yet to be determined, as induction of L4P reporter plasmid transcription by Ad5 genome cotransfection is more efficient than induction by a cocktail of all the activators so far defined. Finally, the L4P was also found to be negatively regulated by its products (17).

The cellular transcription factor TFII-I was initially considered to be a general transcription factor following *in vitro* studies that demonstrated its ability to bind the initiator element (Inr) of promoters and to support basal transcription (18). Later, it was found that TFII-I could function with TBP to drive transcription in promoters that lacked a TATA box, with the Inr element in such promoters directing initiation to a fixed site, similar to the action of the TATA box when present (19). However, subsequent studies have shown that, while TFII-I can direct assembly of the core transcriptional machinery at selected promoters, it is in fact a multifunctional transcription factor with six potential DNA-binding domains of the helix-loop-helix (HLH) class that is able to bind to several unrelated specific DNA sequences (20, 21). TFII-I can act as either an activator or repressor: it has been implicated in transforming growth factor β (TGF-β), B cell activation, and stress response signaling pathways (22–24), while *in vitro* studies have highlighted an interaction with c-Myc at Inr sequences leading to direct transcriptional repression (25).

TFII-I is subject to posttranslational modifications that are likely to be important for its function as a transcription factor. It has been reported to be phosphorylated upon activation of the B cell receptor and by endoplasmic reticulum (ER) stress, leading to its nuclear translocation (23, 26). TFII-I is also a target for posttranslational modification by the adenovirus E4 Orf3 protein. Using a high-throughput screen of SUMO3-conjugated proteins during infection by wild-type (WT) or E4 Orf3 mutant Ad5, Sohn and colleagues identified TFII-I as the major target that was modified in an E4 Orf3-dependent manner (27). The functional significance of these modifications in the context of infection, however, remains to be elucidated.

In this study, we have further characterized the nature of the L4P and its regulation by viral and cellular factors. Having identified the L4P 5′ transcription start site (TSS) by 5′ rapid amplification of cDNA ends (RACE), we observed a potential Inr element at the site. However, mutational analysis showed that although this Inr element bound TFII-I, it was inhibitory for the L4P. The viral L4P activator, E4 Orf3, was also found to work via this Inr element, correlating with its ability to cause changes in TFII-I. Finally, we found that L4-33K, rather than L4-22K, is the more potent negative feedback regulator of the L4P.

## MATERIALS AND METHODS

### Viruses and plasmids.

Infections were carried out with wild-type Ad5 *wt*300 or the E4 Orf3 mutant *in*Orf3 (28) at a multiplicity of infection (MOI) of 10 focus-forming units (FFU) per cell. The Ad5 genomic clone pTG3602-Ad5wt has been described previously (29). Expression plasmids pMEPCMV-IVa2 (30), pcDNA3.1Orf3, and an L103A mutant (31), pcDNA-p53 (32), pE1A and pA-22KFLAG (16), and pCMV22KFLAG and pCMV33KFLAG (7) have been described previously; pcDNAHisLacZ was obtained from Invitrogen and pCI-Neo from Promega. L4P luciferase reporter plasmids based on the pGL3-Basic vector (Promega) were previously described (16). Mutations in the core promoter were created by a two-stage PCR protocol using plasmid L4P 26018-26098Luc as the template, while mutations in extended L4P constructs were constructed by the Quikchange PCR mutagenesis method (Stratagene) using either L4P 25887-26125Luc reporter or pA-22KFLAG plasmid (16) as the template. pCI-22KFLAG and pCI-33KFLAG plasmids were constructed by PCR amplification of the coding sequences of pCMV22KFLAG and pCMV33KFLAG, including the FLAG epitope, using forward and reverse primers containing NheI and EcoRI restriction sites, respectively; PCR products were cloned into pCI-Neo using these sites. pCI-33K-Δds was constructed using two-step PCR mutagenesis and pCI-33K-S192A by the QuikChange method, with pCI-33KFLAG as the template. All primer sequences used for mutagenesis and cloning are available on request.

### siRNA and antibodies.

p53 knockdown was achieved as described previously (17) using validated small interfering RNA (siRNA) (Ambion s605; sense strand, 5′-GUAAUCUACUGGGACGGAATT). The control siRNA was designed, using the siRNA target finder, to have no sequence specificity for any Homo sapiens or Ad5 sequence (Ambion; sense strand, 5′-GAGCCGGACGGCCAAAGAAAUU). Western blot protein detection used the following antibodies at the indicated dilutions: 1:10,000 mouse anti-p53 (DO-1; Santa Cruz), 1:10,000 rabbit anti-Ad late proteins (AdJLB1) (8), 1:500 rat anti-E4 Orf3 (6A11) (33), 1:25,000 mouse anti-glyceraldehyde-3-phosphate dehydrogenase (GAPDH) (GA1R; Thermo Scientific), 1:10,000 rabbit anti-TFII-I (H58; Santa Cruz), and 1:10,000 mouse anti-FLAG (M2; Sigma). The horseradish peroxidase (HRP)-conjugated secondary antibodies were 1:10,000 goat anti-mouse IgG or goat anti-rat IgG (Sigma) and 1:100,000 goat anti-rabbit IgG (Santa Cruz). Chromatin immunoprecipitation (ChIP) used antibodies to TFII-I as described above and normal rabbit IgG (Santa Cruz).

### Cell culture, drug treatment, and transfection.

All cells were maintained at 37°C, 5% CO_2_ in Dulbecco's modified Eagle medium (DMEM) supplemented with 10% fetal calf serum (FCS). All cells were seeded at the appropriate density 24 h prior to the respective procedure. Plasmid and virus DNA transfections were carried out using Transit-LT1 (Mirus) according to the manufacturer's instructions at a ratio of 2 μl reagent per μg of DNA. Where appropriate, DNA transfections were standardized within an experiment to a constant amount of DNA per well using empty vector. siRNA transfections were performed using Lipofectamine 2000 (Invitrogen) according to the manufacturer's instructions, using a ratio of 1 μl reagent per 25 pmol siRNA to achieve a 50 nM final siRNA concentration.

### Luciferase assays.

Luciferase reporter assays were performed essentially as previously described (17). Briefly, cells were harvested in 1× passive lysis buffer (Promega) according to the manufacturer's instructions. The lysates were subjected to analysis for luciferase activity (Bright-Glo; Promega) and β-galactosidase (β-Gal) activity, which served as a control for transfection efficiency. Luciferase activity was normalized to β-galactosidase activity in each sample to generate the relative luciferase activity (RLA). Within a given experiment, RLAs were expressed as fold activity relative to the respective control. All transfections were performed in triplicate.

### SDS-PAGE and Western blotting.

Whole-cell lysates were harvested from 24-well cultures in 1× sample buffer and boiled for 10 min. Typically, 5 to 10% of the sample was separated by SDS-PAGE. Proteins were transferred onto Hybond-ECL nitrocellulose membranes (GE Healthcare), and Western blot analyses were performed using HRP-conjugated secondary antibodies and SuperSignal West Femto Maximum Sensitivity chemiluminescence reagent (Thermo Scientific) as previously described (34).

### RNA extraction and RT-qPCR.

Total RNA was harvested from cells using a GenElute Mammalian Total RNA Miniprep kit (Sigma). RNA was first treated with DNase I (Promega), and then, 500 ng of DNA-free RNA was subjected to reverse transcription (RT) using the GoScript Reverse Transcription system (Promega) with oligo(dT) or random hexamer primers according to the manufacturer's instructions. Typically, 25 to 50 ng of cDNA was used for quantitative-PCR (qPCR) amplification analysis with Brilliant III Ultrafast SYBR green Mastermix (Agilent Technologies) on an MX3005P thermocycler (Stratagene) using universal cycling conditions. The manufacturer's software was used for quantitation of cDNA, either by absolute copy number analysis with a standard curve or by the relative cycle threshold quantitation (ΔΔ*C_T_*) method, according to the experimental requirements. Primers were used at a standard final concentration of 200 nM, and all primer sets were validated for efficiency and specificity using standard curve and melting curve analyses, respectively. For L4-100K and -22K mRNAs, the primers were the following Ad5 genome positions (5′ end; length): forward (26361; 20 nucleotides [nt]) and reverse (26530; 20 nt). For L4-100K, -33K, and -22K, they were as follows: forward (26197; 20 nt) and reverse (26295; 21 nt). For LacZ, they were as follows: 5′-GAAAGCTGGCTACAGGAAG and 5′-GCAGCAACGACGTCA. β-Actin primers were taken from reference 35.

### 5′ RACE and identification of PCR products.

For identification of the the L4P transcription start site, we utilized the FirstChoice RLM-RACE kit (Life Technologies) according to the manufacturer's instructions. Essentially, 1 μg total RNA was treated with calf intestine alkaline phosphatase to remove the 5′ phosphate group of RNA molecules lacking a 5′ cap. The 5′ cap of the mRNA was subsequently removed through the addition of tobacco acid pyrophosphatase, thus exposing a 5′ phosphate group to which an RNA adaptor was ligated using T4 RNA ligase. Reverse transcription was then performed on the ligated RNA using random hexamer primers and Moloney murine leukemia virus (M-MLV) reverse transcriptase. cDNA was subjected to nested PCR using two forward primers complementary to the 5′ adaptor sequence and two reverse primers complementary to the shared L4-22K and L4-33K mRNA sequence [5′ position (length)]: 26782 (24 nt) and 26530 (20 nt). PCRs were performed with GoTaq Hot Start Polymerase (Promega) using the following cycling conditions: 94°C for 1 min; 35 cycles of 94°C for 30 s, 60°C for 30 s, and 72°C for 30 s; and a final extension of 72°C for 7 min. Selected PCR products were excised from the agarose gel, purified using the GeneJet gel extraction kit (Thermo Scientific), and cloned into the pGEM-T Easy vector (Promega), and individual clones were subjected to DNA-sequencing analysis.

### ChIP.

Chromatin immunoprecipitations were performed as previously described (17), except that the sonication step was performed on ice in a Bioruptor (Diogenode) using four 10-min sets of 30-s pulse, 30-s pause on the high setting. One microgram of antibody was used for each immunoprecipitation. The immunoprecipitated DNA was purified using two rounds of phenol-chloroform extraction and subjected to qPCR analysis. The L4P primers were as follows (5′ position; length): forward (25887; 24 nt) and reverse (26072; 20 nt). The percentage of input was calculated by comparing the *C_T_* value of immunoprecipitated DNA with that of an aliquot of the total input DNA.

## RESULTS

### Identification of the 5′ transcription start site of L4-22K and -33K.

**FIG 1:**
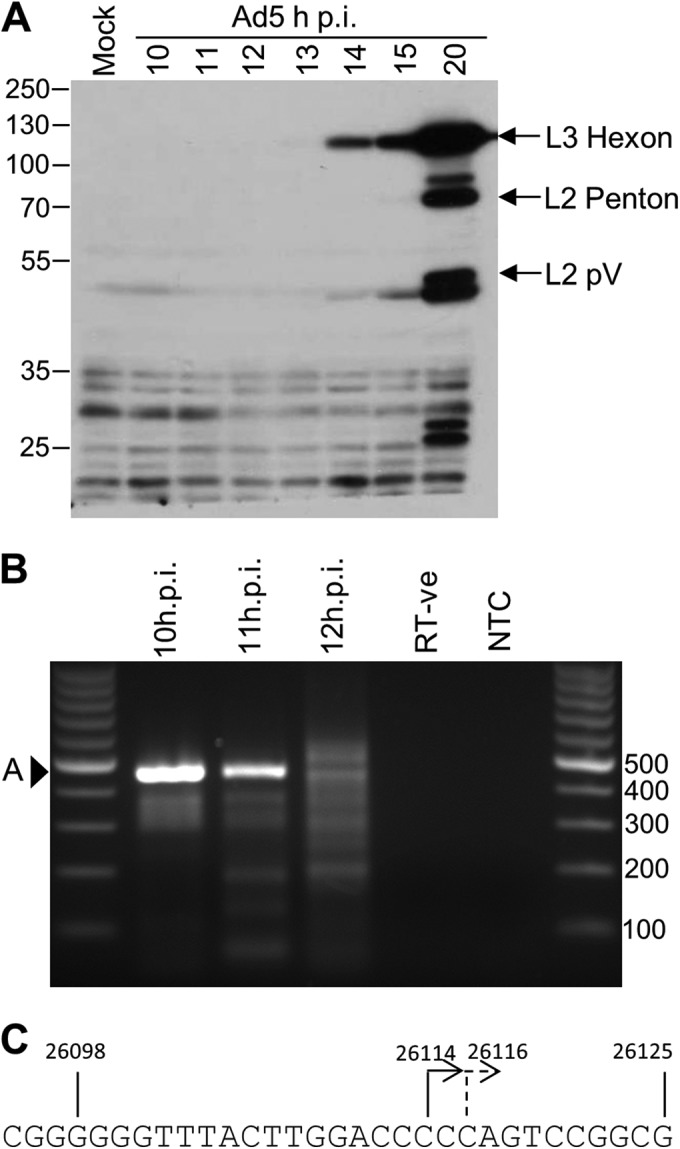
Identification of the 5′ transcription start site of the L4 promoter. (A) HeLa cells were mock infected or infected at a multiplicity of infection of 10 FFU/cell for the times indicated before total protein lysates were harvested. The proteins were separated by SDS-PAGE and subjected to Western blotting with anti-late Ad5 protein antibody. The major bands are identified on the right of the blot; protein sizes are indicated in kilodaltons on the left. (B) HeLa cells were infected as for panel A, and total RNA was harvested at the time points indicated. The RNA was subjected to 5′ RACE and nested PCR using 3′ primers corresponding to the common L4-22K and L4-33K sequence. (C) The PCR products in the 10- and 11-h p.i. samples (panel B, arrowhead A) were excised, cloned, and sequenced. The diagram shows the positions on the Ad5 sequence of the 5′ ends of a series of 14 independent cDNA clones; major and minor start sites are indicated by solid and dashed arrows, respectively. All the numbers indicate nucleotide positions in the Ad5 genome.

Previous studies found that the crucial activating sequences of the L4P lie within nucleotides 25887 to 26125 of the Ad5 genome (16). However, the 5′ transcription start site for L4-22K and L4-33K mRNAs expressed from this promoter remained unknown. We therefore sought to identify the site by employing a 5′ RACE strategy. We first determined a suitable time in infection for the analysis, when L4-22K and -33K transcription was being driven primarily by the L4P rather than the MLP, by analyzing Ad5 late gene expression over a time course in HeLa cells (Fig. 1A). In agreement with our previous studies (17), late protein expression was first detectable at 14 h postinfection (p.i.). Since the proteins detected are expressed under the control of the MLP, the MLP was clearly significantly active by this time p.i., and therefore, it was likely that L4P activity would be diminished by this time. Therefore, we predicted that the L4P was likely to be most active between 10 and 12 h p.i., and thus, we carried out 5′ RACE studies using RNA isolated from infected HeLa cells at these times. To ensure specificity of amplification for authentic L4-22K and L4-33K messages, we employed a nested-PCR approach using 3′ reverse primers located in the cDNA sequence of the 5′ exon common to both proteins. Analysis at 10 h p.i. gave a single predominant PCR product (Fig. 1B, band A) that was also readily detectable at 11 h p.i., though with weaker intensity. By 12 h p.i., this PCR product was no longer the predominant species, and several additional PCR products with similar intensities were detected Sequence analysis of DNA cloned from band A at 10 h p.i. revealed it to be derived from genuine L4-22K and -33K mRNAs, with 100% of the clones defining a TSS that mapped to position 26114 in the Ad5 genome (Fig. 1C). Analysis of band A from the 11-h p.i. sample similarly showed it to be derived from L4-22K and -33K mRNA; 50% of the clones gave this same transcription start site, while the remainder mapped at position 26116 (Fig. 1C). Therefore, these data indicate that transcription from the L4P primarily initiates at position 26114, defined as +1, with an alternative start site at position 26116.

### Validation of extended L4P reporter constructs.

**FIG 2:**
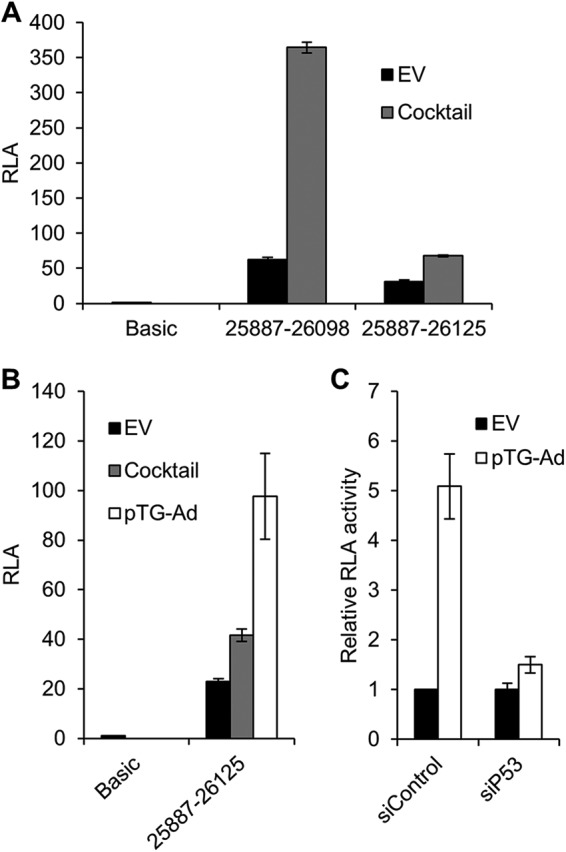
Inducibility of L4P constructs. (A) 293 cells were transfected with a promoterless control plasmid (pGL3-Basic) or the L4P reporter constructs indicated, together with E1A, p53, IVa2, and Orf3 expression plasmids (Cocktail) or an equivalent amount of empty vector (EV). Cells were harvested 24 h later, and reporter gene activity was assayed. RLA, relative luciferase activity, i.e., reporter activity relative to β-Gal activity expressed from a constant amount of pcDNAHisLacZ included as a transfection control. The error bars show the standard deviations from the mean of three replicate samples. (B) 293 cells were transfected with reporter and expression plasmids as for panel A in comparison with a reporter cotransfected with the linearized adenovirus genome (pTG-Ad) or EV; other details are as in panel B. (C) U2OS cells were transfected with 50 nmol of control (siControl) or p53 (siP53) siRNA and 24 h later transfected with L4P 25887-26125 reporter plasmid, together with the linearized adenovirus genome or EV. Samples were collected and assayed as for panel A. The calculated mean RLA values were normalized to each respective siRNA transfection with EV.

Our previous studies to determine the regulatory sequences of the L4P responsive to p53 focused on the region 25887 to 26098 (17), based on observations that it contained elements sufficient for maximal activation. However, in light of the data mapping the L4P transcription start site to a position downstream of this region, we next examined how an L4P luciferase reporter construct that incorporated the natural TSS responded to known activators in comparison with the 25887-to-26098 construct. Upon transfection of 293 cells with these constructs, as expected, both gave significantly higher levels of basal activity (50-fold and 25-fold, respectively) than a promoterless control (16). Upon cotransfection of a cocktail of plasmids encoding established L4P activators IVa2, E4 Orf3, E1A, and p53, activity of the 25887-to-26098 L4P construct was stimulated approximately 7-fold. In contrast, only 2-fold stimulation was achieved for the 25887-to-26125 L4P construct (Fig. 2A).

We previously observed that the cotransfected adenovirus genome was a more potent inducer of L4P 25887-to-26098 activity than the cocktail of activators (17), and therefore, we asked whether the same phenomenon applied to our extended construct (Fig. 2B). While the cocktail of activators led to only a 1.8-fold increase in L4P activity, similar to that shown in Fig. 2A, the Ad5 genome led to a more robust 5-fold increase. We also confirmed that 25887-to-26125 L4P activity is, as previously reported (17), dependent on the cellular tumor suppressor protein p53; siRNA knockdown of p53 almost completely abrogated the stimulation of the L4P by the cotransfected Ad5 genome (Fig. 2C). Altogether, these data demonstrate that an extended L4P reporter construct incorporating the natural L4P TSS is inducible by the same viral and cellular factors that were previously identified as regulators of the L4P. The natural L4P is less strongly activated than the shorter construct by the previously defined cocktail of inducers. This probably reflects a greater dependence of the natural L4P on additional factors arising during infection, since its activation by the cotransfected Ad genome was much greater than that with the plasmid cocktail.

### Architecture of the L4P.

**FIG 3:**
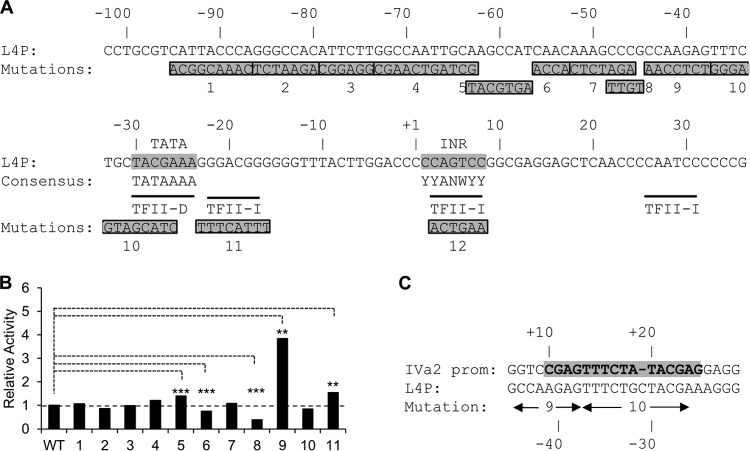
Architecture of the L4 promoter. (A) Sequence of the L4P showing the positions of selected potential transcription factor binding sites, detected using Promo (http://alggen.lsi.upc.es/cgi-bin/promo_v3/promo/promoinit.cgi?dirDB=TF_8.3; TRANSFAC database version 8.3) constrained to human factors and sites and with a dissimilarity cutoff of 10%, and the positions of a set of substitution mutations in the L4P. Matches to the consensus sequences for a TATA box and an initiator element (INR) (38) are indicated by gray shading in the sequence. (B) U2OS cells were transfected with WT or 26018-to-26098 mutant L4P luciferase reporter constructs. RLA values, calculated as for Fig. 2, were normalized to the activity of the WT promoter included within each experiment. The data shown are the means of at least 6 values from at least two independent experiments, except for mutations 4 and 5, which are the means of three values in a single experiment. The significance of differences from the WT was tested by Student's *t* test (two tailed; unpaired; unequal variance): **, *P* < 0.01; ***, *P* < 0.001. (C) Alignment of the known repressor binding site in the Ad5 IVa2 promoter (prom) with a sequence found in the L4P.

At an early stage in our characterization of the L4P, a series of substitution mutations was constructed that covered the core promoter (26018 to 26098) defined by Morris et al. (16) (Fig. 3A). Analysis of the basal activity of each of these mutated promoters in U2OS cells suggested that mutations 6 and 8 reduced activity, suggesting that they affect binding sites for positive factors, while mutations 5, 9, and 11 increased it (Fig. 3B). Having identified the L4P TSS at Ad5 genome position 26114, we also analyzed the L4P region for potential promoter elements *in silico* (Fig. 3A). Only a subset of eukaryotic promoters initiate transcription at a discrete position, and such positioning typically depends on a TATA box located at −25 to −30 relative to the TSS and/or an initiator element (Inr) positioned at the TSS (36, 37). A strong match was found at the L4P TSS to the Inr consensus (YYANWYY) (38), and a weaker predicted TFII-D binding site (TATA box) was detected at −30. Mutation 10 impinges on the TFII-D site but had only a small negative effect on L4P activity that was not significant. Mutation 9 gave the strongest evidence for a repressor site within the core promoter; this mutation alters a sequence that is a close match to the binding site for the cellular repressor of IVa2 gene expression defined by Flint and colleagues (Fig. 3C) (39–41); while mutation 10 also impacts this sequence, its effect on the overlapping TATA box would be expected to be dominant. The activation of the L4P occurs at the same phase of infection as the activation of the IVa2 promoter, and thus, it might be expected that there would be common features in their regulation, such as the IVa2 repressor, but we have not explored this possibility further.

### The Inr element of the L4P contains a repressor-binding site for TFII-I.

**FIG 4:**
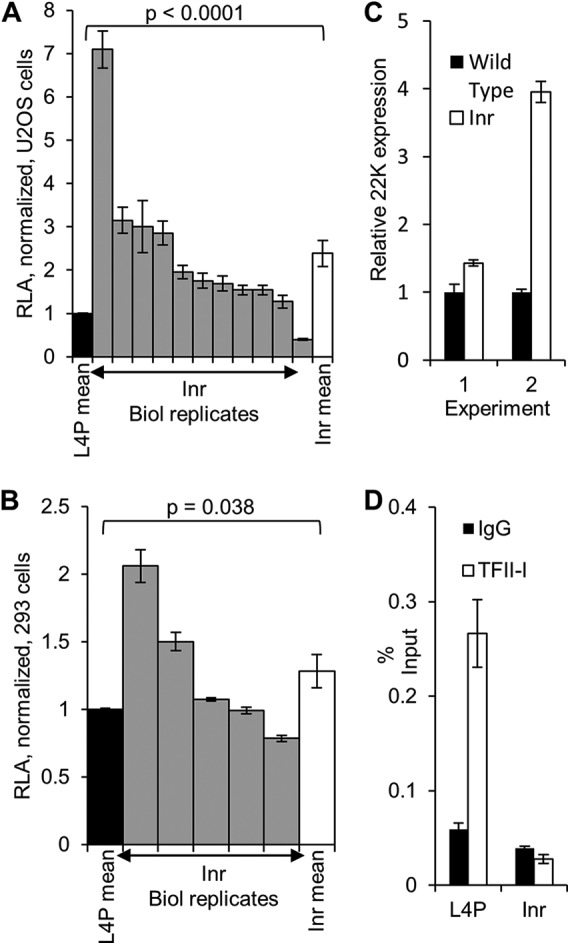
TFII-I inhibits the L4P through the Inr sequence. (A) U2OS cells were transfected with WT or Inr 25887-to-26125 mutant L4P luciferase reporter constructs, and 24 h later, cells were harvested, reporter gene activity was assayed, and RLA was calculated as for Fig. 2. The individual mean Inr mutant activities from each of 11 independent experiments conducted at different times are displayed (gray bars); the error bars show the standard deviations of three replicate samples in each experiment. The mean activities of the WT and Inr mutant L4P constructs across the 11 biological (Biol) replicates are displayed by the black and white bars, respectively; the error bars indicate the standard errors of the mean. The *P* value was obtained using Student's *t* test (two-tailed; unequal variance), comparing the WT and Inr mean activities. (B) Same as panel A, except that five biological-replicate experiments were performed in 293 cells. (C) 293 cells were cotransfected with WT or Inr mutant pA-22KFLAG expression plasmid (16), together with pcDNAHisLacZ plasmid. After 24 h, total RNA was harvested and subjected to RT-qPCR analyses for 22K and LacZ mRNAs. The relative expression was calculated using the ΔΔ*C_T_* method, using LacZ expression for normalization. The data shown are the means of three technical replicates and are from two independent experiments; the error bars indicate the standard deviations. (D) 293 cells were transfected with wild-type or Inr 25887-to-26125 mutant L4P luciferase reporter plasmids. Twenty-four hours later, cells were harvested for ChIP analysis using control IgG or TFII-I antibodies, and immunoprecipitated L4P DNA was quantified by qPCR analysis. The data are presented as percent input DNA, which was measured in parallel from a sample of extract reserved prior to immunoprecipitation, and are shown as the means of three technical replicates; the error bars indicate the standard deviations.

Both the location of the putative Inr sequence at the L4P TSS and its high level of similarity to the consensus for such elements suggested that it might be functionally significant. To investigate this, we mutated the predicted Inr sequence within the 25887-to-26125 L4P reporter construct (Fig. 3A, mutation 12) and assayed its basal activity in 293 and U2OS cells (Fig. 4A and B). To our surprise, given the expectation of a positive role for the Inr element in L4P activity, analysis in U2OS cells revealed the basal activity of the Inr mutant to be very significantly higher than that of the wild type, though considerable variation was seen between independent experiments (Fig. 4A). Analysis in 293 cells also revealed the basal activity of the Inr mutant to be significantly greater than that of the wild type, though to a lesser extent and again with a wide variance among determinations (Fig. 4B).

To confirm this effect, we used an alternative system to measure the basal activity of the Inr mutant L4P. The pA-22KFLAG plasmid contains Ad5 genomic sequence from nucleotide 25887 through the L4P TSS and the complete L4-22K coding region, concluding with an added C-terminal FLAG epitope cDNA sequence (16), and so represents the L4P in its most natural context. Consistent with our reporter assays, a version of pA-22KFLAG containing a mutated Inr sequence displayed increased basal activity over the wild type in 293 cells (Fig. 4C), though as before, there was considerable variation between experiments.

These data suggested that the Inr element within the L4P may contain a repressor-binding site. Inr elements are reported to bind the cellular transcription factor TFII-I, which has been demonstrated to possess both transcriptional activation and repression abilities (21, 42). In fact, three binding sites for TFII-I were predicted in the vicinity of the TSS by our *in silico* analysis (Fig. 3A), one of which was positioned over the Inr element. Of the other two sites, the upstream site was in inverted orientation and covered by mutation 11 while the downstream site lay outside the functional promoter fragment 25887 to 26125 and was not considered further. Mutation 11 increased basal activity somewhat in the core promoter (Fig. 3B) and by 2.7-fold in the extended promoter background (not shown), suggesting that TFII-I might also act as an inhibitor via this site. We therefore attempted to detect association of endogenous TFII-I with the L4P using chromatin immunoprecipitation on the WT or the Inr 25887-to-26125 mutant L4P reporter construct (Fig. 4D). Following plasmid transfection into 293 cells, which we used because of their greater transfection efficiency and higher level of endogenous TFII-I than U2OS cells, the L4P could indeed be specifically precipitated by a TFII-I antibody and, consistent with our hypothesis, mutation of the Inr element reduced TFII-I binding to background levels. Similar results were obtained in U2OS cells (data not shown). Taken together, these data suggest that TFII-I associates with the L4P via the Inr site and inhibits L4P activity through association with this site.

### E4 Orf3 activates the L4P via its effect on TFII-I.

**FIG 5:**
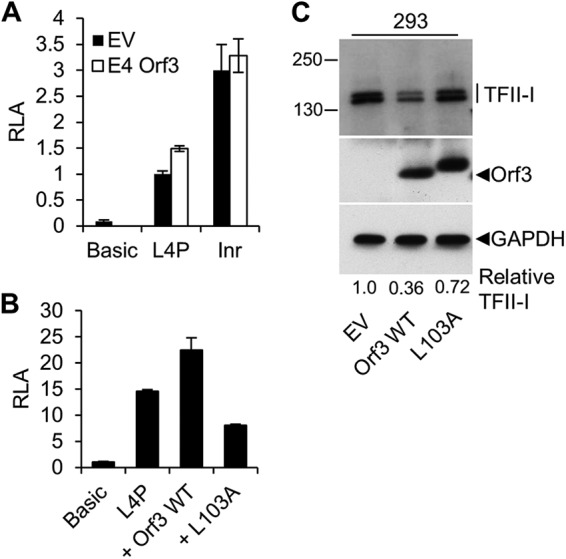
E4 Orf3 regulates the L4P through TFII-I. (A) U2OS cells were cotransfected with a promoterless control plasmid (Basic) or a wild-type (L4P) or Inr mutant L4P luciferase reporter plasmid, together with an E4 Orf3 expression plasmid or EV. Twenty-four hours later, cells were harvested and reporter gene activity was assayed as for Fig. 2. The data shown are the means of a minimum of five replicate samples across two independent biological experiments; the error bars indicate the standard deviations from the mean. (B) Same as panel A, but using only a wild-type L4P reporter and additionally testing the transactivation ability of L103A mutant Orf3. (C) 293 cells were transfected with WT or mutant Orf3 plasmids, and after 24 h, total protein lysates were harvested, separated by SDS-PAGE, and subjected to Western blotting with anti-TFII-I, anti-Orf3, or anti-GAPDH antibody. Protein sizes are indicated in kilodaltons. L103A Orf3 protein reproducibly migrates more slowly than the wild type (31, 43). The band intensities in the digitized images were quantified using QuantityOne software (Bio-Rad); the amounts of TFII-I detected, relative to the GAPDH control, are shown below the blots.

A recent study by Sohn and coworkers demonstrated that in Ad5-infected HeLa cells, TFII-I is extensively posttranslationally modified by sumoylation, dependent on expression of the viral E4 Orf3 protein (27). These findings suggested a possible link between our observation of the negative regulation of the L4P by TFII-I and our previous finding that E4 Orf3 was an L4P activator (16). If E4 Orf3 activation of the L4P was mediated via its effect on TFII-I, then activation would have to depend on the presence of the TFII-I binding site. To test this, we compared the ability of E4 Orf3 to activate wild-type and Inr mutant L4P reporter plasmids (Fig. 5A). As observed previously, the basal activity of the Inr mutant was significantly higher than that of the wild type. Upon addition of E4 Orf3, the activity of the wild-type L4P was stimulated by approximately 1.5-fold, whereas no activation of the Inr mutant L4P was observed. Thus, E4 Orf3 activation of the L4P requires the Inr element, supporting the idea that E4 Orf3 acts on the L4P via its effects on TFII-I. To further test this idea, we compared the properties of wild-type E4 Orf3 with those of the L103A mutant (shown previously to lack all the described Orf3 functions, including oligomerization [43]). Comparable expression of the wild type and the L103A mutant was achieved (Fig. 5B); as observed previously (31, 43), the L103A mutant migrated more slowly than the wild-type protein. While wild-type E4 Orf3 activated the L4P as before, L103A Orf3 failed to do so (Fig. 5B). Interestingly, while wild-type Orf3 expression caused a reduction in detectable TFII-I, similar to that seen by Sohn et al. during Ad5 infection (27), L103A Orf3 lacked this ability (Fig. 5C); 293 cells were used here because of their superior transfection efficiency. Thus, the ability of E4 Orf3 to activate the L4P correlates with its ability to affect TFII-I, as detected by Western blotting. Taken together with the dependence of Orf3 activation of the L4P on TFII-I binding at the Inr site, these data strongly suggest that Orf3 activates the L4P by disrupting an inhibitory activity of TFII-I.

### Regulation of the L4P by negative feedback depends on L4-33K.

**FIG 6:**
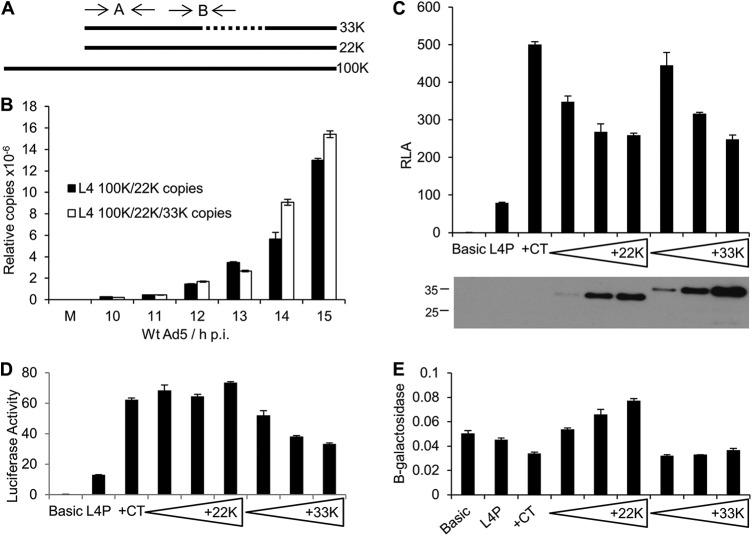
L4-33K is a negative regulator of L4P activity. (A) Schematic of the Ad5 L4 region displaying the approximate positions of RT-qPCR primers used for panel B. Primer pair A detects total L4-22K, -33K, and -100K mRNA, whereas primer pair B detects L4-22K and -100K mRNA only. (B) HeLa cells were infected with wild-type Ad5 at an MOI of 10 FFU per cell for the times indicated before total RNA was harvested and subjected to RT-qPCR analysis with the primer sets indicated in panel A. mRNA copy numbers were calculated using a standard curve generated using L4-22K plasmid and normalized between samples to the respective actin *C_T_* values. (C) (Top) 293 cells were cotransfected with promoterless control plasmid (Basic) or 25887-to-26125 WT L4P reporter construct alone (L4P) or together with a cocktail of p53, E1A, and E4 Orf3 expression plasmids (+CT) supplemented with either empty vector or increasing amounts of pCI-22KFLAG or pCI-33KFLAG.Twenty-four hours later, cells were harvested and reporter gene activity was assayed as for Fig. 2. The data shown are the means of three replicate samples; the error bars indicate the standard deviations. (Bottom) One replicate well from each of the transfections indicated was subjected to SDS-PAGE and Western blotting with anti-FLAG antibodies to confirm 22K or 33K protein expression; protein sizes are indicated in kilodaltons. (D) Luciferase reporter activities from panel C without normalization. (E) β-Galactosidase values from panel C.

The L4P is likely to be active in only a brief window during Ad5 infection, as it rapidly becomes part of the MLTU and its activity is subject to negative feedback by its products (17). It has been reported recently that L4-22K is necessary for the accumulation of mature L4-33K mRNA (15), implying that L4-22K must accumulate before L4-33K during the infection process. We therefore hypothesized that transcription from the L4P produces primarily L4-22K mRNA. To address this question, we quantified L4-22K and L4-33K transcripts from wild-type Ad5-infected cells at various times postinfection. Initial attempts to utilize an L4-33K-specific primer set were unsuccessful due to significant cross-amplification of L4-22K template (data not shown). Due to the genetic architecture of the L4 region, any primers directed at L4-22K would also amplify message encoding L4-100K. Therefore, we opted instead to compare the amount of L4-100K, -22K, and -33K mRNA with that of L4-100K and -22K mRNA using RT-qPCR with the primer pairs illustrated in Fig. 6A, with the difference between the signals detected reflecting the amount of L4-33K mRNA relative to other L4 mRNAs. Analysis of infected-cell RNA showed that from 10 to 13 h p.i. the amounts of RNA detected by the two amplicons were very similar (the small excess of amplicon B at 13 h is probably explained by the accumulation of variant RNAs lacking the primer sites for amplicon A), indicating that there was little or no L4-33K mRNA present at this time. However, from 14 h p.i., the amount of L4-100K, -22K, and -33K mRNA exceeded that of L4-100K and -22K mRNA, suggesting that L4-33K mRNA was now being produced (Fig. 6B). As noted above, this timing coincides with the onset of expression from the MLP (Fig. 1A). These data support the notion that the L4P primarily drives expression of L4-22K, with L4-33K mRNA being produced only subsequently.

The relative timing of L4-22K and L4-33K expression presented the logic that L4-33K should be the primary mediator of L4P negative-feedback control previously observed using 25887-to-26098 constructs (17). We therefore analyzed the effects of L4-22K and L4-33K expression on the activity of an L4P reporter plasmid construct containing the natural transcription start site. IVa2 was omitted from the activating cocktail for these experiments, as it is not required for inhibition by L4-22K and -33K (17) and it is a known binding partner for L4-22K (10) that might therefore affect L4-22K activity indirectly. Activation of the 25887-to-26125 L4P reporter in 293 cells by a cocktail of p53, E1A, and E4 Orf3 expression plasmids led to the expected 5-fold increase in L4P activity (Fig. 6C). Addition of either L4-22K or L4-33K apparently led to a dose-dependent inhibition of L4P activity (Fig. 6C); the fact that this did not reach background levels is likely due to the limitations of getting effective cotransfection of all cells with the two plasmids at achievable levels of DNA input. However, closer inspection of the data showed that only the L4-33K effect was due to a genuine inhibition of luciferase expression from the L4P reporter (Fig. 6D); the apparent reduction in L4P activity upon L4-22K expression was due solely to a dose-dependent increase in expression from the β-galactosidase transfection control plasmid (Fig. 6E), with L4P luciferase activity remaining constant (Fig. 6D). These data suggested that only L4-33K has a specific inhibitory effect on the activity of the L4P.

**FIG 7:**
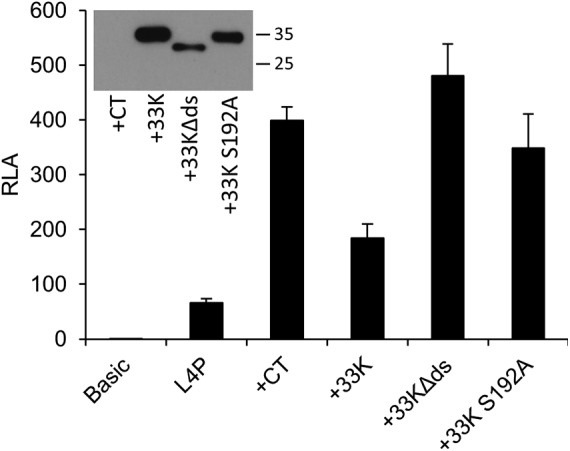
293 cells were transfected with promoterless control plasmid (Basic) or 25887-to-26125 L4P reporter plasmid alone (L4P) or together with a cocktail of p53, E1A, and E4 Orf3 expression plasmids (+CT) supplemented with either empty vector (+CT) or pCI-33KFLAG wild-type (+33K), 33KΔds, or 33K S192A expression plasmid. After 24 h, cells were harvested and reporter gene activity was assayed as for Fig. 2. The data shown are the means of three replicate values; the error bars indicate the standard deviations. (Inset) One replicate well from each of the transfections indicated was subjected to SDS-PAGE and Western blotting with anti-FLAG antibodies to confirm 33K protein expression; protein sizes are indicated in kilodaltons.

In order to validate this conclusion further, we constructed two mutations in L4-33KFLAG, both located in the conserved C-terminal domain of the protein that is not shared with L4-22K. 33KΔds corresponds to a rare, natural 33K splice variant that specifies a 27-amino-acid in-frame deletion that removes an RS repeat region, while S192A targets one RS element within that region, as previously described by Törmänen et al. (13). Both mutations destroy L4-33K function in late mRNA splicing and disrupt the localization of the protein to replication centers during adenovirus infection (13, 44). We tested the abilities of these mutated L4-33K proteins to inhibit L4P activation by a cocktail of activators containing p53, E1A, and E4 Orf3 (Fig. 7). Addition of the cocktail led to a 6-fold increase in L4P activity, and addition of wild-type 33K reduced this activation by 50%, similar to that shown in Fig. 6C. However, neither 33KΔds nor S192A L4-33K was able to inhibit L4P activity. Thus, the L4P-inhibitory activity of L4-33K depends on a domain of the protein that is not shared with L4-22K, further supporting the conclusion that L4-33K has a unique inhibitory effect on the L4P.

## DISCUSSION

The L4P plays a pivotal role in the replication cycle of Ad5, with its products serving both to activate the MLP and to drive the correct pattern of alternative mRNA processing from the MLTU; both of these actions are essential to the formation of viral structural proteins in the correct amounts and proportions. Here, we have identified the L4P 5′ transcription start site and, based on this knowledge, used a reporter plasmid strategy to define a putative initiation (Inr) element within the L4P whose activity within the promoter was repressive rather than activating. In common with other Inr elements, the L4P Inr mediated binding of the cellular factor TFII-I to the promoter. The previously defined the viral L4P activator, E4 Orf3, was found also to work via the Inr element, its positive effect correlating with its ability to cause a change in TFII-I. Finally, we reexamined negative-feedback regulation of the L4P and found that L4-33K is the principal effector of this control.

The early understanding of eukaryotic transcription focused on the idea of a fixed site of initiation that was associated with an upstream TATA box, but it is now clear that this model fits only a minority of promoters. Constitutively active promoters more typically initiate over a broad region, while even among promoters that have a defined TSS, only around 20% have a recognizable TATA box (37). A number of other sequence elements that characterize the cores of such promoters have been identified, including Inr elements at the TSS. Such elements have been shown to cooperate with, or even to replace, the TATA box in the recruitment of the essential basal transcription factor TFII-D to the promoter (45).

In our initial *in silico* review of the L4P, the most prominent potential core promoter feature was a putative Inr element at the TSS. However, the start sites we mapped did not correspond to the typical position for promoters in which the TSS is fixed by a functional Inr element. In such promoters, initiation normally occurs on the almost invariant A rather than on upstream pyrimidines, as in the L4P, although the TSS position can vary around an Inr element depending on the strength and position of the Inr relative to the TATA box (46). In the L4P, the major TSS is 30 nucleotides downstream of a putative TATA box, the canonical distance, and its position is therefore more likely to be determined by the TATA box than by the Inr. This interpretation fits with our earlier observation of strong promoter activity conferred by 25887 to 26098, a fragment that contains the putative TATA box but lacks the Inr (16). Also supporting this conclusion, our *in silico* analysis detected a second, equally good match to the Inr consensus at +24, but no initiation was detected at this position. Another feature typically found in promoters displaying a discrete start site that lack a TATA box is a downstream promoter element (DPE); with the consensus RGWYV, this element is found at +28/32 to the TSS and is a secondary binding site for TFII-D (45). L4P position +28/32 lacks any plausible match to the DPE consensus, further supporting the conclusion that the L4P TSS is positioned primarily by its TATA box, even though this would be expected to be a relatively weak feature based on its sequence.

Mutation of the putative L4P Inr element increased basal promoter activity and abolished TFII-I binding to the promoter, defining TFII-I as an inhibitor of the promoter via this element. We also observed that mutation of a second putative TFII-I binding site located at −20 similarly increased L4P basal activity. The fact that mutating just one of the two sites was sufficient to disrupt TFII-I binding suggests that inhibition of the L4P by TFII-I requires both sites to be intact. The extent of the activity advantage of the Inr mutant over the WT promoter varied considerably between experiments. We believe that the most likely explanation for this is that transfection delivers different amounts of reporter plasmid to cells in the various experiments, leading to the TFII-I inhibitory activity being “titrated out” to different extents and, hence, altering the extent of increased activity of the Inr mutation versus the WT. Alternatively, the level of this TFII-I inhibitory activity may fluctuate with the biology of the cell cultures over time.

The inhibitory effect of TFII-I on the L4P Inr element contrasts with data for the Ad5 MLP, where TFII-I binding to an Inr element was shown previously to increase basal activity *in vitro* (18) and to synergize with USF in MLP activation *in vivo* (47). However, current understanding of the functional significance of TFII-I interactions with Inr elements is incomplete, since these elements have been reported also to bind other proteins, including TFII-D (45), and they may also play a role in Inr function. Thus, depending on its sequence, each Inr element may recruit different combinations of proteins in which the effect of TFII-I on promoter activity can vary. TFII-I is also structurally and functionally heterogeneous. There are at least four isoforms, of which TFII-Iα, -β, and -Δ appear to be ubiquitously expressed whereas TFII-Iγ is restricted to neuronal cells, and different isoforms may exert opposite effects on a given promoter, e.g., the murine c-*fos* gene is subject to opposing actions of the β and Δ isoforms (21, 42, 48). Each isoform also contains multiple potential DNA-binding domains, interaction with which may not always confer the same function. It is possible, therefore, that the differences in the effects of TFII-I on the L4P and MLP reflect differences in the relative affinities of activating and inhibitory isoforms or domains for the two sequences.

In our original characterization of the L4P, we showed that the Ad5 E4 Orf3 protein upregulated the promoter. The same protein was also shown recently to greatly increase the number of sumoylation sites in TFII-I during Ad5 infection (27). In the present study, we found that E4 Orf3 activation of the L4P operated via the Inr site and correlated with the ability of Orf3 to cause modification of TFII-I, as detected in our hands by a reduction of TFII-I on Western blots. This effect on TFII-I is likely to be related to the reduction in unmodified TFII-I at 7 h p.i. in HeLa cells that was observed by Sohn et al., concomitant with substantially enhanced TFII-I sumoylation (27). Sumoylation is known both to generate and to disrupt protein-protein interactions (49). Thus, the substantial modification of TFII-I during infection would be predicted to have major effects on its function, in particular its participation in transcriptional complexes and its binding to Inr elements. Sohn et al. also reported that the presence of Orf3 altered the subcellular localization of TFII-I, recruiting it into Orf3 structures in the nucleus (27). This, too, would be expected to alter or to prevent the interaction of TFII-I with DNA target sites.

The suggestion that TFII-I might be inactivated by E4 Orf3 during the early-intermediate phase of infection may seem paradoxical, given that TFII-I has been ascribed a positive role in MLP activity and the promoter is strongly active in the late phase of infection. However, significant mutations in the MLP Inr are compatible with normal levels of late mRNA and virus yield when the canonical strong MLP TATA box is present (50), and there is no strong evidence for a major role for TFII-I in MLP activation *in vivo* (51), so a loss of TFII-I activity during the late phase is not incompatible with MLP activity. Equally, it is possible that the form of TFII-I that is inhibitory to the L4P and that is inactivated by E4 Orf3 is distinct from the TFII-I that has the potential to activate MLP or that E4 Orf3-enhanced sumoylation of TFII-I selectively inhibits only some TFII-I functions.

L4P activity produces RNA that could encode either L4-22K or L4-33K, depending on its pattern of splicing. We found that in the critical period during infection when the L4P was most active, the L4 RNA present showed little evidence of splicing to produce 33K-encoding mRNA, suggesting that the L4P expresses primarily 22K. This fits with the previous report that L4-22K is necessary for L4-33K expression (15). We further found that of the two proteins, L4-33K had the more significant activity as a repressor of the full-length L4P construct and that this activity depended on a sequence within the C-terminal domain of the protein that is highly conserved among human adenoviruses.

In its splicing-regulatory function, L4-33K interacts with an RNA splicing enhancer element (for IIIa splicing, designated 3VDE) in the MLTU transcript (13). This interaction involves the C-terminal domain unique to 33K that includes a short SR region reminiscent of other splicing-regulatory factors. Mutating one particular serine residue in this domain to glycine abolished splicing enhancer activity and L4-33K localization to Ad5 replication centers (13, 44), and we found that mutating the same residue to alanine was also sufficient to abolish negative regulation of the L4P. This suggests that the functional bases of the two activities are similar, raising interesting questions about the mechanism by which L4-33K inhibits the L4P. Possibly, the C-terminal domain of L4-33K can also bind to specific DNA sequences to mediate its effect on the L4P. Alternatively, it might act via binding to the nascent L4P RNA transcript, although, since it inhibits expression from reporter constructs that have very little Ad5 sequence downstream of the TSS, such an interaction could not be highly sequence specific. Finally, L4-33K might inhibit the L4P by interfering with the action of one or more protein activators of the promoter, though in previous work we showed that none of the defined activators of the L4P was a unique target of L4-33K inhibition and that IVa2, a potential binding partner of L4-33K (52), was not required for inhibition (17).

It is possible that inhibitions of the L4P by TFII-I and L4-33K are related phenomena, though such a scenario is unlikely for two reasons. First, TFII-I is sufficient for inhibition of the L4P in the absence of L4-33K, as our luciferase reporter studies were performed in the absence of any L4 gene products. Second, we have demonstrated that L4-33K accumulates only once activation of the L4P is well under way, suggesting that by this time repression of the L4P by TFII-I has been efficiently relieved by E4 Orf3. However, it is possible that L4-33K is required to recruit TFII-I back onto the L4P at later times of infection. Further studies would be needed to explore this possibility and to test directly the effects of these regulators on the L4P in the context of virus infection.

In conclusion, our new data shed further light on the regulation of the activity of the Ad5 L4P during the intermediate phase of the infectious cycle that signifies the transition from early to late patterns of gene expression. The present understanding of the activity of the L4P can be summarized as follows. A combination of viral and cellular factors exert both positive and negative regulation of the promoter, leading to a brief transient wave of activity that provides principally the L4-22K protein; this protein has been shown to act on the MLP and on MLTU RNA processing (7, 11, 12, 15). One of the effects of L4-22K is to cause expression of the L4-33K protein (15), which, with L4-22K, ensures the correct and complete pattern of late gene expression from the MLTU that is essential for a full productive infection while, as we show here, inhibiting the L4P. The effect of the cell environment on the activity of cellular regulators of the L4P, including TFII-I (this study) and p53 (17), is likely to be an important determinant of the outcome of infection.
